# The Effectiveness and Safety of Probiotic Supplements for Psoriasis: A Systematic Review and Meta-Analysis of Randomized Controlled Trials and Preclinical Trials

**DOI:** 10.1155/2021/7552546

**Published:** 2021-12-13

**Authors:** Liuting Zeng, Ganpeng Yu, Yang Wu, Wensa Hao, Hua Chen

**Affiliations:** ^1^Department of Rheumatology and Clinical Immunology, Peking Union Medical College Hospital, Chinese Academy of Medical Sciences & Peking Union Medical College, National Clinical Research Center for Dermatologic and Immunologic Diseases (NCRC-DID), Key Laboratory of Rheumatology and Clinical Immunology, Ministry of Education, Beijing, China; ^2^People's Hospital of Ningxiang City, Ningxiang City, Hunan Province, China; ^3^Institute of Material Medical, Chinese Academy of Medical Sciences & Peking Union Medical College Institute of Materia Medica, Beijing, China

## Abstract

**Background:**

Patients with psoriasis need long-term medication to control their condition. Recent studies suggest that changing the intestinal flora may be a potential treatment.

**Methods:**

The databases were utilized to search the randomized controlled trials (RCTs) and preclinical trials about probiotic supplement in the treatment of psoriasis. The retrieval time is from the establishment of these databases to December 2020. RevMan5.3 was used for the risk assessment of bias and meta-analysis. This systematic review was registered in PROSPERO (CRD42021232756).

**Results:**

A total of 3 RCTs involving 164 participants were included. Two RCTs showed that probiotics can improve PASI and thereby improve the condition. For inflammation-related indicators, only one RCT showed that probiotics can improve the levels of CRP and TNF-*α* but have no obvious improvement effect on IL6. One RCT demonstrated the total effective rate of probiotics in the treatment of psoriasis. For adverse events, one RCT showed that the incidence of adverse events of probiotic treatment was low. Preclinical studies showed that continuous intervention with oral probiotics can significantly improve the progression of psoriasis and reduce the expression of inflammatory factors. The meta-analysis showed that the PASI between two groups was of no statistical significance (SMD 1.83 [-0.41, 4.07], *P* = 0.11). Meanwhile, probiotics may improve skin thickness (SMD -5.87 [-11.34, -0.41], *P* = 0.04) in animal model.

**Conclusion:**

Prebiotics may have a positive effect on alleviating the clinical symptoms of psoriasis, but a large sample of RCTs is still needed to support its therapeutic effect in psoriasis.

## 1. Introduction

Psoriasis is a chronic inflammatory skin disease, which has a long course and tends to recur easily. The incidence of this disease is mainly young and middle-aged, and it has a greater impact on the physical health and mental status of patients. Epidemiology shows that the global prevalence among adults and children is 2-3% and 0.5-1%, respectively [1, 2], and the prevalence rate reported in the United States in 1996 is 2.6% [2]. The clinical manifestations are mainly erythema and scaly, which can be affected by the whole body. The scalp and the extensor side of the limbs are more common, and most of them worsen in winter. The course of the disease is longer, and patients are prone to relapse [3]. At present, patients with psoriasis need long-term medication to control their condition. There are many clinical treatment methods, including narrow-band ultraviolet radiation phototherapy, oral acitretin, *Tripterygium wilfordii*, methotrexate, and cyclosporine. Methotrexate and etanercept are often used in China, and recombinant human interleukin, infliximab, adalimumab, ustekinumab, secukinumab, etc. are also used abroad [4–7]. However, side effects such as gastrointestinal reactions and liver and kidney toxicity of these drugs lead to poor patient compliance. In addition, due to the high cost of some biological agents, patients' access to medicines is restricted. Therefore, it is urgent to explore new supplements and alternative treatment options.

Recent studies suggest that the inflammatory microenvironment formed by bacterial products, intestinal immune cell migration, and systemic cytokine release may cause psoriasis [8, 9]. The diversity of intestinal microbiota in stool samples of patients with psoriasis is significantly reduced, and a variety of digestive tract bacteria are generally reduced in patients with psoriasis and psoriatic arthritis. Hence, changing the intestinal flora may be a potential treatment [8, 9]. The current research also shows that it has certain advantages to improve the symptoms and pathological process of psoriasis by adjusting and improving the intestinal microbial community strategy of psoriasis [10, 11]. Methods to improve the regulation of microbial communities include probiotics, prebiotics, and fecal bacteria transplantation [12–14]. At present, preclinical trials and clinical trials of probiotics for the treatment of psoriasis continue to appear. They showed that probiotics can reduce the psoriasis area and severity index of psoriasis patients, inhibit the inflammation level of psoriasis, regulate immune cells, and regulate the composition of the microbiota [15–17]. However, there are still many deficiencies in related studies, and there is a lack of a summary of the above-mentioned research reports and an evaluation of the level of evidence of the above-mentioned studies. Meanwhile, some clinical evidence reports are insufficient in sample size estimation, blind method, and methodological quality evaluation. Therefore, a systematic review of the effectiveness and safety of clinical studies and preclinical studies of probiotics in the treatment of psoriasis is imminent. Therefore, this study hopes to conduct a systematic review of randomized controlled trials (RCTs) of probiotics in the treatment of psoriasis and summarize the clinical effects of probiotics in the treatment of psoriasis, in order to provide guidance for clinical practice and provide reference for future RCTs of probiotics to treat psoriasis.

## 2. Materials and Methods

### 2.1. Protocol

This systematic review and meta-analysis were conducted strictly in accordance with the protocol registered in PROSPERO (CRD42021232756) and PRISMA-guidelines (see supplementary materials).

### 2.2. Selection Criteria

For RCTs, they were considered eligible if they met the PICO criteria: (1) participants: the patient was diagnosed as an adult patient with psoriasis by a doctor with sufficient clinical qualifications according to the clinical diagnostic criteria for psoriasis. For psoriasis, there is no limit to first diagnosis or recurrence; (2) intervention: the intervention of the experimental group is probiotic preparations, with no restriction on bacteria species, probiotic content, etc. The intervention of the control group is a placebo or other nonprobiotic intervention methods; (3) outcomes: Psoriasis Area and Severity Index (PASI), inflammation-related indicators, total effective rate, adverse events; (4) study design: RCTs; and (5) exclusion criteria: non-RCTs, non-adult patients.

For animal experiments, preclinical studies were considered eligible if they met the following criteria: (1) animal population: rats or mice, no restriction on strain, age, and so on; (2) intervention: the intervention of the experimental group is probiotic preparations, with no restriction on bacteria species, probiotic content, etc. The intervention of the control group is blank or other nonprobiotic intervention methods; (3) outcomes: improvement of skin damage, inflammatory immune factors, immune cell composition, etc.; (4) and exclusion criteria: absence of a matched control group and the full text is not available.

### 2.3. Literature Search Strategy

For clinical trials, we searched the English database (Web of Science, Medline, PubMed, and Embase) and Chinese database (China National Knowledge Infrastructure (CNKI), Wanfang Database, VIP Database for Chinese Technical Periodicals, and China Biology Medicine (CBM)). The retrieval time is from the establishment of these databases to 16 December 2020. In addition, we also searched the Cochrane Library (to Issue 12, 2020) and ClinicalTrials.gov. The search strategy of PubMed and Embase is shown in Table 1 as an example.

For animal experiments, we searched the Web of Science, Medline, PubMed, Embase, CNKI, Wanfang Database, VIP Database for Chinese Technical Periodicals, and China Biology Medicine (CBM). The retrieval time is from the establishment of these databases to 16 December 2020. The search strategy of PubMed and Embase is shown in Table 2 as an example.

### 2.4. Data Extraction and Risk of Bias Assessment

The selection of literature and data extraction were carried out independently by two researchers according to the data extraction table established in advance. The results will be cross-checked after completing the literature selection and data extraction. If the two researchers have a disagreement, they will discuss whether to include or exclude the literature. If they cannot reach a consistent conclusion, they will discuss with all the researchers to resolve it. The data extraction table includes the name of the first author, publication time, country, scale, intervention measures, and outcomes.

The literature quality evaluation adopts Cochrane Collaboration's risk of bias evaluation standard. It mainly includes the following: (1) random sequence generation method; (2) whether to hide the allocation; (3) blind method; (4) whether the outcome data is complete; (5) whether there is a selective report; and (6) other biases. The quality of the literature was evaluated independently by two researchers. If there is a disagreement, the decision is made through discussion with all researchers.

### 2.5. Statistical Analysis

The Review Manager 5.3 software was used for statistical analysis. For continuous variables, the standard mean difference (SMD) was used to describe the effect size, and the confidence interval (CI) is 95%. The *χ*^2^ test was used to analyze the heterogeneity between the results. In the case of low heterogeneity (*P* > 0.1, *I*^2^ < 50%), a fixed effects model analysis was performed. If there is heterogeneity between the studies, a random effects model is used.

## 3. Results

### 3.1. Results of the Search

**Figure 1:**
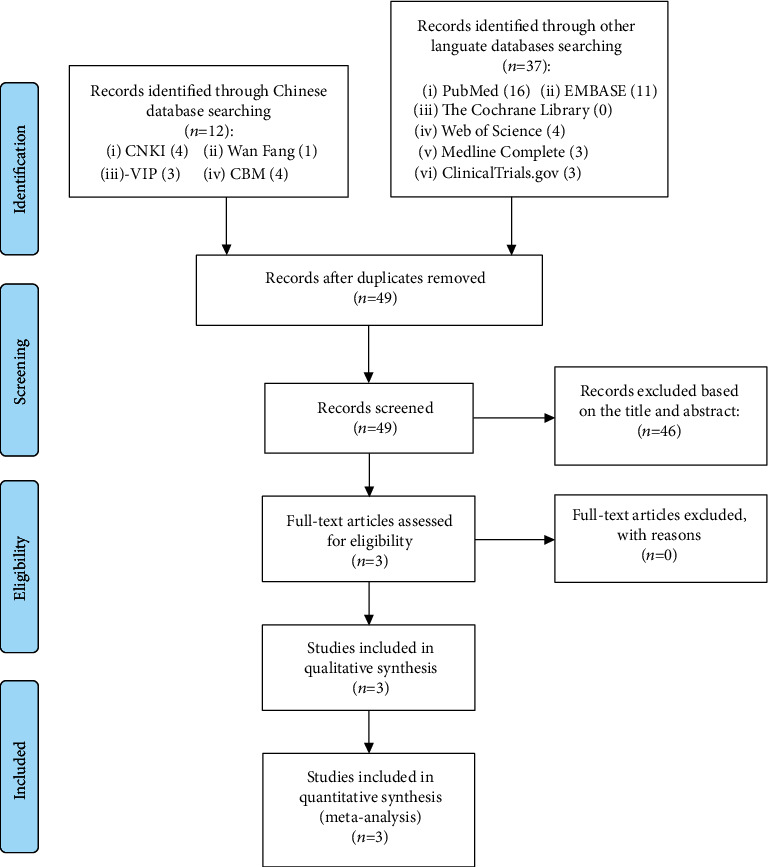
Flow diagram for searching RCT.

The total records identified through database searching and other sources were 49. According to the search strategy, a total of 3 articles were obtained through preliminary search. By eliminating duplicate documents, carefully reading the title and abstract, a total of 46 articles were excluded. After carefully reading the full text and comparing the selection criteria, 3 RCTs were screened out and finally included [15–17] (Figure 1).

**Figure 2:**
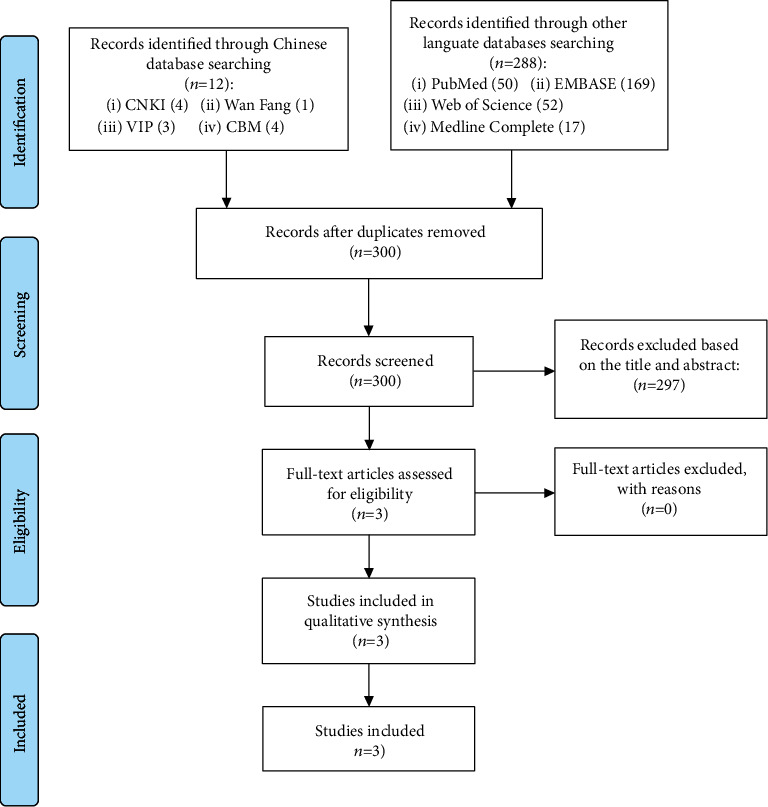
Flow diagram for searching animal experiments.

Regarding preclinical research, we initially retrieved a total of 300 articles from 8 databases. After screening, 3 articles that may meet the conditions were obtained, which were subsequently retained. After reading the full text, three eligible animal model research articles were finally included to analyze the effect of probiotic intervention on psoriasis (Figure 2).

### 3.2. Description of Included Trials

The 3 RCTs are all from different countries, and the research scale is about 20-90 participants. The publication year of the included RCTs is 2013-2019. The intervention measures of the 3 RCTs are all probiotics, but the sources of probiotics are different. The details of study characteristics are presented in Table 3.

### 3.3. Risk of Bias of Included Studies

**Figure 3:**
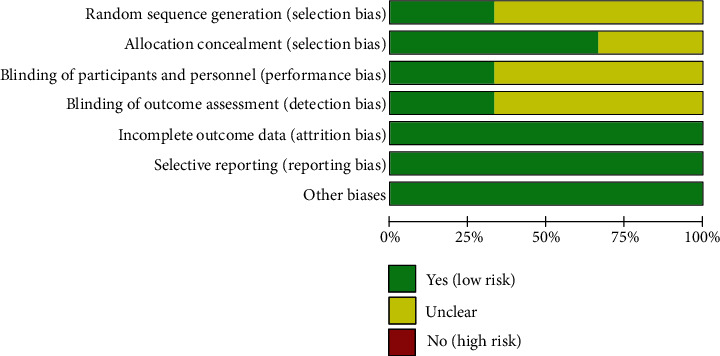
Risk of bias graph.

**Figure 4:**
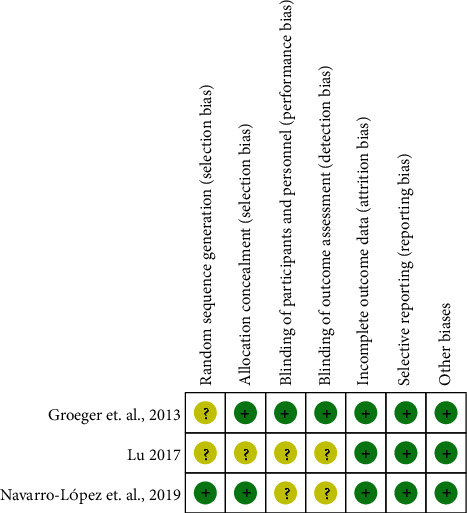
Risk of bias summary.

The summary and graph of risk of bias are shown in Figures 3 and 4.

#### 3.3.1. Sequence Generation and Allocation Concealment

Only Navarro-López et al. [15] described the method of random sequence generation, which is a computer-generated random sequence. Therefore, it is assessed as low risk of bias. The other two RCTs did not describe the method of random sequence generation and were therefore assessed as unclear risk of bias.

Lu [17] did not describe whether to use allocation concealment, so it was rated as unclear risk of bias. The remaining two RCTs used similar packaging for the tablets of the test group and the control group. They were considered to have adopted allocation concealment and therefore were assessed as low risk of bias.

#### 3.3.2. Blinding, Incomplete Outcome Data and Selective Reporting

Navarro-López et al. [15] and Lu [17] claimed that they used blinding but did not describe the process of blinding implementation, so they were rated as unclear risk of bias. Groeger et al. [16] described the blinding of both patients and researchers and was therefore rated as low risk of bias.

Although there are missing data in Navarro-López et al. [15], the intention-to-treat analysis was used, so it was rated as low risk of bias. Groeger et al. [16] and Lu [17] did not observe incomplete outcomes, so they were rated as low risk of bias. All RCTs do not have selective reporting and are therefore considered a low risk of bias.

#### 3.3.3. Other Potential Bias

Other sources of bias were not observed in 4 RCTs; therefore, the risks of other bias of the RCTs were low.

### 3.4. PASI

**Figure 5:**
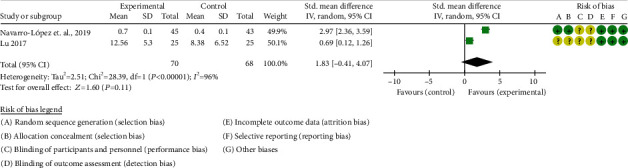
The results of PASI.

Two RCTs reported PASI [15, 17]. Due to their different description methods (Navarro-López et al. use percentage improvement to describe PASI, while Lu directly gives PASI score); they cannot be combined for statistical analysis. The results of Navarro-López et al. showed that the improvement of PASI in the probiotic group was better than that in the placebo group (*P* = 0.03). Lu also showed that PASI improved better after probiotic intervention (probiotic vs. placebo: 6.82 ± 4.22 vs. 10.76 ± 5.35; *P* < 0.05). The results were taken as absolute values and statistically analyzed. The summary results showed that the PASI between two groups was of no statistical significance (SMD 1.83 [-0.41, 4.07], *P* = 0.11; random effects model) (Figure 5). The funnel chart shows that the possibility of publication bias might be low (Figure S1).

### 3.5. CRP, TNF-*α*, and IL-6

Only Groeger et al. [16] reported CRP, TNF-*α*, and IL-6. Compared with control group, CRP (*P* = 0.0425) and TNF-*α* (*P* = 0.0405) were reduced after the intervention of probiotics. For IL-6, the study showed that there was no significant difference between the probiotics intervention and the placebo group (*P* > 0.05).

### 3.6. Total Effective Rate

Only Lu [17] reported the total effective rate: Total effective rate = the number of cases of (cure + effective + improvement)/total number of cases × 100%. Efficacy index = (PASI total score before treatment − PASI total score after treatment)/PASI before treatment; cure: curative effect index > 95%; effective: curative effect index 60%-95%; improved: curative effect index 30%~<60%; and ineffective: curative effect index < 30%. Lu showed that in the probiotic group, 14 were cured, 4 were effective, 5 were improved, and 2 were ineffective; the total effective rate was 92%. In the control group, 12 were cured, 4 were effective, 2 were improved, and 7 were ineffective, with a total effective rate of 72%. The difference between the probiotic group and the control group was statistically significant (*P* < 0.05).

### 3.7. Adverse Events

Only Navarro-López et al. [15] reported adverse events. Navarro-López et al. showed a low incidence of adverse events, and no patients withdrew from treatment due to adverse reactions (no specific data shown). It also showed that no serious adverse events occurred in both groups.

### 3.8. Evidence Quality Assessment

To promote the conclusion, the GRADE tool was utilized to rate the quality of the evidence [18]. According to the GRADE handbook [19], the evidence was judged to be very low (Table 4).

### 3.9. Animal Studies

This study finally included 3 preclinical studies based on animal models (Table 5). Three studies used different probiotics to treat IMQ-induced psoriasis-like skin inflammation model. Chen et al. [20] found that oral administration of *L. pentosus* GMNL-77 may decrease erythematous scaling lesions. It may also decrease the expression of TNF-alpha, IL-6, IL-23, IL-17A/F, and IL-22 mRNA and the number of Th17/Th22 T cells. Rather et al. [21] found that SEL001 may improve skin lesions and pathological changes and decrease vertical skin thickness. SEL001 may also decrease the expression of IL-19, IL-17A, and IL-23 mRNA. Wang et al. [22] found that *Escherichia coli* Nissle 1917 improves skin lesions and pathological changes and decreases vertical skin thickness. *Escherichia coli* Nissle 1917 may decrease the serum IL-8, IL-23, IL-10, and TNF-*α* levels and increase the serum IL-10 level; it may also decrease the expression of IL-17A, IL-17F, IL-23, TNF-*α* mRNA, and increase the expression of IL-10 mRNA.

**Figure 6:**
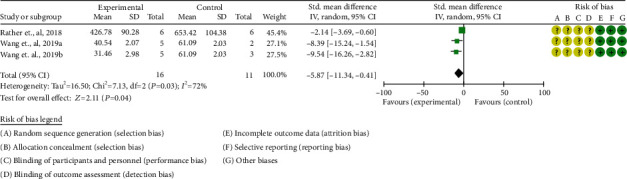
Skin thickness.

In general, the above-mentioned preclinical animal model studies have shown that continuous intervention with oral probiotics can significantly improve the progression of psoriasis and reduce the expression of inflammatory factors. According to the preliminary results of animal studies, probiotics may have a certain regulatory effect on immune response, inflammatory response, and immune cell composition. The skin thickness of the animal was analyzed statistically. The summary results showed that probiotics can improve skin thickness (SMD -5.87 [-11.34, -0.41], *P* = 0.04; random effects model), suggesting that it can improve psoriasis skin lesions (Figure 6).

## 4. Discussion

### 4.1. Main Outcomes Summary

The data of two RCTs reporting PASI supports that probiotics can improve PASI and thereby improve the condition. For inflammation-related indicators CRP, TNF-*α*, and IL-6, only one RCT showed that probiotics can improve the levels of CRP and TNF-*α* but have no obvious improvement effect on IL6. Lu's study demonstrated the total effective rate of probiotics in the treatment of psoriasis. He defined the treatment result as follows: cure: curative effect index > 95%; effective: curative effect index 60%-95%; improved: curative effect index 30%~<60%; and ineffective: curative effect index < 30%. Total effective rate = the number of cases of (cure + effective + improvement)/total number of cases × 100%. Lu showed that in the probiotic group, 14 were cured, 4 were effective, 5 were improved, and 2 were ineffective; the total effective rate was 92%. In the control group, 12 were cured, 4 were effective, 2 were improved, and 7 were ineffective, with a total effective rate of 72%. His research shows that probiotics are more effective in treating psoriasis. Regarding the safety of probiotic therapy, only Navarro-López et al. reported adverse events. Their RCT showed that the incidence of adverse events of probiotic treatment was low, no serious adverse events occurred, and no patients fell off due to adverse events.

### 4.2. Applicability of Evidences

Psoriasis is a chronic inflammatory skin disease characterized by erythema. The causes of psoriasis are complex, different patients' conditions are quite different, and patients are prone to disease reactions during treatment [23, 24]. At present, many modern studies believe that a variety of factors, such as patients' genetic factors, infections, metabolic disorders, endocrine disorders, neuropsychiatric factors, and patients' immune disorders, may be the cause of psoriasis [25]. At present, there is no safe, effective, and long-term treatment for psoriasis. In recent years, the incidence of psoriasis has risen sharply, especially among young patients. The results of the psoriasis epidemiological survey report show that in my country, the incidence of psoriasis disease is 0.123%. The treatment of scoria mainly depends on the treatment of drugs such as acitretin or the treatment of narrow-band ultraviolet rays [26]. There are many clinical studies on acitretin, and the results of many studies have confirmed that acitretin has a good effect on psoriasis. However, acitretin may cause a variety of adverse reactions. This results in poor compliance of patients with acitretin, especially in the treatment of young patients with psoriasis [27]. It may cause adverse reactions, such as skin itching, conjunctivitis, neurological symptoms, musculoskeletal pain, and fatigue, and affect the normal work and life of the patient [28]. As a result, the patient cannot take the medication on time according to the doctor's order, and it is easy to cause the patient to stop the drug by himself due to the occurrence of the adverse reaction during the treatment or the patient has to stop the drug treatment due to the serious adverse reaction. In addition, there are patients who have other health problems caused by taking acitretin, which affects their health. Therefore, the use of acitretin in clinical practice is restricted [29, 30]. In addition, due to the effects of acitretin on the female reproductive system, acitretin is a contraindicated drug for pregnant women, breastfeeding women, and patients who are planning to have children in the near future [31]. These have also affected the widespread use of acitretin in clinical practice. In addition, due to the occurrence of adverse reactions in the clinic, the dose of acitretin is reduced or the patient can not adhere to the medication, etc., resulting in a significant decrease in the efficacy of acitretin in the treatment of psoriasis [32]. In this case, many patients use acitretin for psoriasis that does not have a good therapeutic effect, or they have repeated attacks. Therefore, the development of safer and more effective treatments in clinical practice is an urgent problem to be solved.

At present, the epidemiological survey of patients with psoriasis and gastrointestinal discomfort has found that the incidence of gastrointestinal discomfort and inflammatory bowel disease in patients with psoriasis is higher than that of healthy people, and vice versa. Another study showed that 7% to 11% of patients with inflammatory bowel disease also suffer from psoriasis, which shows that the relationship between psoriasis and gastrointestinal inflammation is particularly close [33–35]. In the study of the intestinal flora of psoriasis and inflammatory bowel disease, it was found that certain beneficial bacteria (such as *Clostridium prasectus*) were reduced in both psoriasis and inflammatory bowel disease [8, 36, 37]. Meanwhile, patients with psoriasis have significantly increased intestinal inflammation-related autoantibodies and inflammatory cell infiltration, similar to those in inflammatory bowel disease [38]. Moreover, the difference in intestinal flora is closely related to the host genotype, and there is a certain cross-over between the susceptibility genes of patients with psoriasis and inflammatory bowel disease [8, 39]. It is inferred from this that psoriasis is related to the systemic inflammatory response and immune problems caused by intestinal flora disorders and that intestinal flora disorders are involved in the systemic lesions associated with psoriasis [8]. On the other hand, certain genetic and environmental factors and immune pathways jointly participate in the pathogenesis of these two diseases. For example, Th17 cells and their cytokines play a major role in the development of psoriasis, and they are also involved in the physiopathological process of inflammatory bowel disease [40]. Intestinal flora can control imiquimod-induced psoriasis skin inflammation by changing T cell responses, which suggests that intestinal flora affects the pathogenesis of psoriasis [41]. This provides another possible explanation for the relationship between psoriasis, inflammatory bowel disease, and intestinal flora.

Existing studies have found that the diversity and relative abundance of the intestinal flora of patients with psoriasis is significantly reduced [42]. Among them, *Bifidobacterium*, *Broutella*, *Faecococcus*, etc. are significantly reduced in the intestinal tract of patients with psoriasis. Meanwhile, the content of short-chain fatty acid (SCFA) in the stool of patients with psoriasis is significantly lower than that of healthy people [43]. SCFA can regulate the number and functions of T cell populations by promoting the induction and fitness of T cells in the colon environment. SCFA can play an irreplaceable role in promoting the immune balance of Treg cells such as Th1/Th2 and Th17/Treg. Insufficiency or deficiency of SCFA is related to cellular energy, nutrient metabolism, physical barriers, and immune inflammatory response [44]. Certain species of Firmicutes can upregulate Treg cells through G protein-coupled receptor 43 (GPR43) of SCFA [45]. T/B lymphocytes, DC cells, macrophages, NK cells, and other immune cells are closely related to the pathogenesis of psoriasis [46]. HUANG et al. used 16S rRNA to sequence the feces of 35 patients with psoriasis and 27 healthy people and found that the psoriasis group and the healthy group had differences in flora [47]. The flora of patients with severe psoriasis is different from the flora of patients with mild psoriasis, and the flora of the healthy control group is also different, which confirms that patients with psoriasis have obvious disordered flora [34]. It can be seen that further research on psoriasis based on the microbiota may provide new insights into the pathogenesis of psoriasis and provide more evidence for the prevention and treatment of psoriasis. In addition, studies have found that the diversity of intestinal flora in patients with psoriasis is significantly reduced, and people with low intestinal flora diversity are more likely to be in a low-grade inflammation state [48, 49]. Short-chain fatty acids (SCFA) are the fiber fermentation products of the intestinal flora and play a key role in promoting the integrity of the intestinal barrier and exerting anti-inflammatory effects. It is mainly derived from Fischer bacillus, but the content of Fischer bacillus in the intestines of patients with psoriasis is significantly lower than that of healthy subjects [50, 51]. Butyric acid is an important component of SCFA, which is mainly secreted by *Clostridium prastigma* of Firmicutes. It is of great significance in promoting the anti-inflammatory effect of SCFA and maintaining the integrity of the intestinal barrier [52]. However, studies have found that the number of *Clostridium plasmodium* in the intestine of patients with psoriasis has been significantly reduced, suggesting that changes in the composition of the intestinal flora of patients with psoriasis may be an important factor in causing immune inflammation [53]. Another study showed that the increase in the ratio of intestinal Firmicutes/Bacteroidetes in patients with psoriasis is the main feature of the psoriasis intestinal flora, which is specifically manifested in the increase in the abundance of Firmicutes and the decrease in the abundance of Bacteroidetes [54, 55]. An increase in the ratio of Firmicutes/Bacteroidetes can affect the metabolism of intestinal carbohydrates and reduce the production of SCFA, which ultimately leads to chronic inflammation and damage to the intestinal barrier. In addition, impaired intestinal barrier function can directly cause bacteria and their metabolites to be released into the blood and promote the occurrence of systemic inflammatory reactions, thereby inducing or aggravating psoriasis [56]. New research shows that the occurrence and development of psoriasis are closely related to the imbalance of intestinal flora, which can promote the progression of psoriasis by increasing the level of immune inflammatory response in the body. Its core mechanism may be related to the release of intestinal metabolites (LPS and glycolipids) into the blood and activation of inflammatory response and immune-related signaling pathways [40, 57].

Probiotics are a class of active microorganisms that produce beneficial effects on the host by regulating the intestinal microecological balance and play an important role in immune regulation, metabolic processes, and neuroendocrine [58]. For a long time, active organisms (probiotics) have been introduced to selectively enhance the intestinal microbiota, or indigestible carbohydrates (prebiotics) have been given to actively promote growth, thereby controlling the intestinal microbiota [59]. The application of prebiotics in atopic dermatitis, acne, and wound healing has achieved good results [60]. Probiotics have an immunomodulatory effect on the skin and enhance the skin barrier repair function by reducing the bacterial load of the skin and antagonizing invasive symbiosis [61]. In a 6-day animal experiment, IRFAN et al. found that *Lactobacillus* probiotic-65 improved the severity of imiquimod-induced psoriasis in mice. It also reduces the expression levels of psoriasis-related proinflammatory cytokines such as IL-17A, IL-19, and IL-23 [21]. It can be considered that *Lactobacillus* can not only relieve clinical symptoms but also reduce the level of proinflammatory cytokines. Chen and other teams also confirmed this view. They evaluated the effect of *Lactobacillus pentosus* GMNL-77 on a mouse model of imiquimod-induced psoriasis. Compared with untreated mice in the control group, mice treated with probiotics had significantly fewer erythema, scales, and thickened epidermis [20]. Magdolna et al. summarized the effects of oral probiotics on psoriasis and found that 3 different probiotics have been shown to improve the condition [62]. However, the existing data is limited and uneven, so it is difficult to propose a program for proper supplementation of probiotics in patients with psoriasis.

### 4.3. Discussion of the Source of Heterogeneity

The heterogeneity of PASI (clinical test results) and skin thickness (animal test results) is high. The high heterogeneity of animal experiment results is mainly due to the different strains, breeding environment, and intervention drugs. The possible reasons for the high heterogeneity of clinical trial outcomes might be as follows: (1) Different RCTs use different probiotic preparations. (2) The populations included in different RCTs are different (the populations involved in the three RCTs are Spanish, Ireland, and China), and there are individual differences. (3) The age groups of patients included in different RCTs are different. (4) Different ways of describing outcomes have caused heterogeneity (Navarro-López et al. use percentage improvement to describe PASI, while Lu directly gives the PASI score).

### 4.4. The Strengths and Limitation of This Review

The strengths of this systematic review and meta-analysis is that this study evaluated the efficacy and safety of probiotics in the treatment of patients with psoriasis for the first time, comprehensively integrating the RCTs and preclinical experimental results of probiotics in the treatment of patients with psoriasis.

The limitation of this review are as follows: (1) Due to the lack of clinical trials of probiotics for the treatment of psoriasis, this study only included 3 RCTs and 3 preclinical studies, involving only 164 participants, which affected the stability and generalization of the results. (2) The inconsistency of probiotic preparations, different age groups of people, different regions, and different result descriptions have led to the heterogeneity of outcomes. (2) The ambiguity of random sequence generation and allocation, the high heterogeneity of outcomes, and the lack of participants lead to the low quality of evidence (GRADE grading is very low).

### 4.5. Implications for Future Research

For future clinical practice, the preparation and types of probiotics are recommended to be unified, and it is recommended to report the outcomes in a unified way (such as directly reporting the endpoint PASI score and endpoint inflammation index value). For basic research, it is recommended to further explore the mechanism of probiotics to interfere with psoriasis in the future and explore the effects of probiotics on the intestinal flora and intestinal metabolism of patients with psoriasis. In short, more well-designed and high-quality RCTs and preclinical studies are still needed in the future to correct the results of this study.

## 5. Conclusion

Prebiotics may have a positive effect on alleviating the clinical symptoms of psoriasis and may be used as a treatment strategy for psoriasis, but in the future, a large sample of RCTs is still needed to support its therapeutic effect in psoriasis.

## Figures and Tables

**Table 1:** Search strategies for PubMed and Embase.

PubMed	Probiotics AND (Psoriasis OR Psoriases OR Pustulosis of Palms and Soles OR Pustulosis Palmaris et Plantaris OR Palmoplantaris Pustulosis OR Pustular Psoriasis of Palms and Soles) AND (random^∗^ controlled trial [pt] OR controlled clinical trial^∗^ [pt] OR randomized [tiab] OR placebo [tiab] OR drug therapy [sh] OR random^∗^ [tiab] OR trial^∗^ [tiab] OR group^∗^ [tiab]) NOT (animals [mh] NOT humans [mh])

EMBASE	1. “Probiotics”/exp 2. “psoriasis”/exp 3. “Pustulosis of Palms and Soles” 4. “Pustulosis Palmaris et Plantaris” 5. “Palmoplantaris Pustulosis” 6. “Pustular Psoriasis of Palms and Soles” 7. 2 or 3 or 4 or 5 or 6 8. “randomized controlled trial” 9. “single blind procedure” or “double blind procedure” 10. “crossover procedure” 11. 8 or 9 or 10 12. 1 and 7 13. 11 and 12

**Table 2:** Search strategies for PubMed and Embase.

PubMed	Probiotics AND (Psoriasis OR Psoriases OR Pustulosis of Palms and Soles OR Pustulosis Palmaris et Plantaris OR Palmoplantaris Pustulosis OR Pustular Psoriasis of Palms and Soles) NOT (humans [mh] NOT animals [mh])

EMBASE	1. “Probiotics”/exp 2. “psoriasis”/exp 3. “Pustulosis of Palms and Soles” 4. “Pustulosis Palmaris et Plantaris” 5. “Palmoplantaris Pustulosis” 6. “Pustular Psoriasis of Palms and Soles” 7. 2 or 3 or 4 or 5 or 6 8. 1 and 7

**Table 3:** The characteristics of the included studies.

Study	Country	Sample size		Intervention		Relevant outcomes	Inclusion criteria	Exclusion criteria	Mean age (years)		Duration
		Trial group	Control group	Trial group	Control group				Trial group	Control group	
Navarro-López et al. 2019 [15]	Spanish	45	43	Oral probiotics (*Bifidobacterium longum* CECT 7347, *B. lactis* CECT 8145, and *Lactobacillus rhamnosus* CECT 8361) + topical corticosteroid betamethasone in combination with calcipotriol	Oral placebo (maltodextrin) + topical corticosteroid betamethasone in combination with calcipotriol	PASI75, adverse events	Age between 18 and 70 years, diagnosis of plaque psoriasis at least one year prior to the study, mild or moderate severity (PASI > 6), and informed consent signed	1. Had exposure to systemic corticosteroids, methotrexate, cyclosporine, or biologic drugs in the previous 3 months 2. Antibiotics in the previous 2 weeks 3. Signs of bacterial infection 4. The diagnosis of liver disease with child-Pugh C index 5. Chronic renal insufficiency with creatinine clearance <50 ml/min or serum creatinine >1.5 mg/dl 6. Chronic endocrine, respiratory, neurological, or moderate to severe cardiovascular disease, as well as concomitant skin disease prior to the start of the study	41.57 ± 13.23	43.09 ± 10.32	12 weeks
Groeger et al. 2013 [16]	Ireland	12	14	Oral probiotics (*Bifidobacterium infantis* 35264)	Oral placebo (maltodextrin)	CRP, TNF-*α* and IL-6	Aged between 18 and 60 y with mild to moderate chronic plaque psoriasis with a Psoriasis Area and Severity Index (PASI) < 16	1. Pregnant or breast feeding females 2. Individuals diagnosed with lactose intolerance or immunodeficiency 3. Individuals who had undergone any abdominal surgery (with the exception of hernia repair and appendectomy) 4. Individuals with a psychiatric illness or with significant hepatic, renal disease	—	—	8 weeks
Lu 2017 [17]	China	25	25	Oral probiotics (combined *Bifidobacterium*, *Lactobacillus*, *Enterococcus*, and *Bacillus*) 1 g bid or tid + oral acitretin 10 mg tid; after 2 months, it was changed to 10 mg bid, and the dose was gradually reduced to 0	Oral acitretin 10 mg tid; after 2 months, it was changed to 10 mg bid, and the dose was gradually reduced to 0	Total effective rate, PASI75	1. The patient meets the diagnosis of psoriasis vulgaris in the “"Guiding Principles for Clinical Research of New Chinese Medicines” 2. Age from 18 to 70 years old 3. The patient or the patient's family voluntarily participate and sign the informed consent form	1. Received treatment for psoriasis within 1 month 2. Unusual psoriasis 3. Severe chronic diseases of the heart, liver, and kidneys or mental system diseases that make it impossible to communicate normally 4. Patients during pregnancy or lactation	51.3 ± 5.6	52.2 ± 5.9	12 weeks

PASI: psoriasis area and severity index; CRP: C-reaction protein; ESR: erythrocyte sedimentation rate.

**Table 4:** Summary of findings for the main comparison.

Outcomes	Illustrative comparative risks (95% CI)^∗^	No of participants (studies)	Quality of the evidence (GRADE)	Comments
PASI	The mean PASI in the intervention groups was 1.83 standard deviations higher (0.41 lower to 4.07 higher)	138 (2 studies)	⊕⊝⊝⊝ very low^1,2,3^	SMD 1.83 (-0.41 to 4.07)

^∗^The basis for the *assumed risk* (e.g., the median control group risk across studies) is provided in footnotes. The *corresponding risk* (and its 95% confidence interval) is based on the assumed risk in the comparison group and the *relative effect* of the intervention (and its 95% CI). CI: confidence interval; GRADE: working group grades of evidence. Very low quality: we are very uncertain about the estimate. ^1^Downgraded one level due to serious risk of bias (random sequence generation, allocation concealment, blinding, and incomplete outcomes), and most of the data comes from the RCTs with moderate risk of bias. ^2^Downgraded one level due to the probably substantial heterogeneity. ^3^Downgraded one level due to the total sample size fails to meet the optimal information size.

**Table 5:** Published studies examining the effects of probiotics supplementation on psoriasis in animals.

Study	Country	Model	Intervention	Measurements	Main findings	Duration
Chen et al. 2017 [20]	Taiwan, China	Imiquimod- (IMQ-) induced psoriasis-like skin inflammation model, BALB/c mice, 6-8 weeks old	G1: control; G2: model; G3: *Lactobacillus pentosus* GMNL-77 (5 × 10^7^ CFU/d); G4: *Lactobacillus pentosus* GMNL-77 (5 × 10^8^ CFU/d)	Skin lesions, inflammatory cytokines, the number of Th17/Th22 T cells	1. Oral administration of *L. pentosus* GMNL-77 may decrease erythematous scaling lesions 2. *L. pentosus* GMNL-77 may decrease the expression of TNF-alpha, IL-6, IL-23, IL-17A/F, and IL-22 mRNA 3. *L. pentosus* GMNL-77 may decrease the number of Th17/Th22 T cells	6 days
Rather et al. 2018 [21]	Korea	IMQ-induced psoriasis-like skin inflammation model, ICR mice, 6 weeks old	G1: control (*n* = 6); G2: model (*n* = 6); G3: *Lactobacillus sakei* Probio65 SEL001 (*n* = 6)	Skin lesions, vertical skin thickness, pathological changes, IL-17A, IL-19, and IL-23 mRNA expression	1. SEL001 may improve skin lesions and decrease vertical skin thickness 2. SEL001 improved pathological changes 3. SEL001 may decrease the expression of IL-19, IL-17A, and IL-23 mRNA	6 days
Wang et al. 2019 [22]	China	IMQ-induced psoriasis-like skin inflammation model, BALB/c mice, 6-8 weeks old	G1: control (*n* = 5); G2: model (*n* = 5); G3: *Escherichia coli* Nissle 1917 low dose (*n* = 5); G4: *Escherichia coli* Nissle 1917 high dose (*n* = 5)	Skin lesions, vertical skin thickness, pathological changes, serum inflammatory factors, Th17/Treg-related factor mRNA	1. *Escherichia coli* Nissle 1917 may improve skin lesions and decrease vertical skin thickness 2. *Escherichia coli* Nissle 1917 improved pathological changes 3. *Escherichia coli* Nissle 1917 may decrease serum IL-8, IL-23, IL-10, and TNF-*α* levels and increase serum IL-10 level 4. *Escherichia coli* Nissle 1917 may decrease the expression of IL-17A, IL-17F, IL-23, and TNF-*α* mRNA and increase the expression of IL-10 mRNA	24 days

## Data Availability

The data used to support the findings of this study are included within the article.

## Abbreviations

RCT: Randomized controlled trial
CNKI: China National Knowledge Infrastructure
CBM: China Biology Medicine
SCFA: Short-chain fatty acid
GPR43: G protein-coupled receptor 43.

## Supplementary Materials

Supplementary Materials PRISMA 2009 checklist: a checklist of systematic review and meta-analysis for RCT. Figure S1: the funnel chart of PASI.
