# Supplementary figures and images for: The Effectiveness and Safety of Probiotic Supplements for Psoriasis: A Systematic Review and Meta-Analysis of Randomized Controlled Trials and Preclinical Trials
Source: J Immunol Res. 2021 Dec 13;2021:7552546. doi: 10.1155/2021/7552546 (PMC8687811; doi:10.1155/2021/7552546)

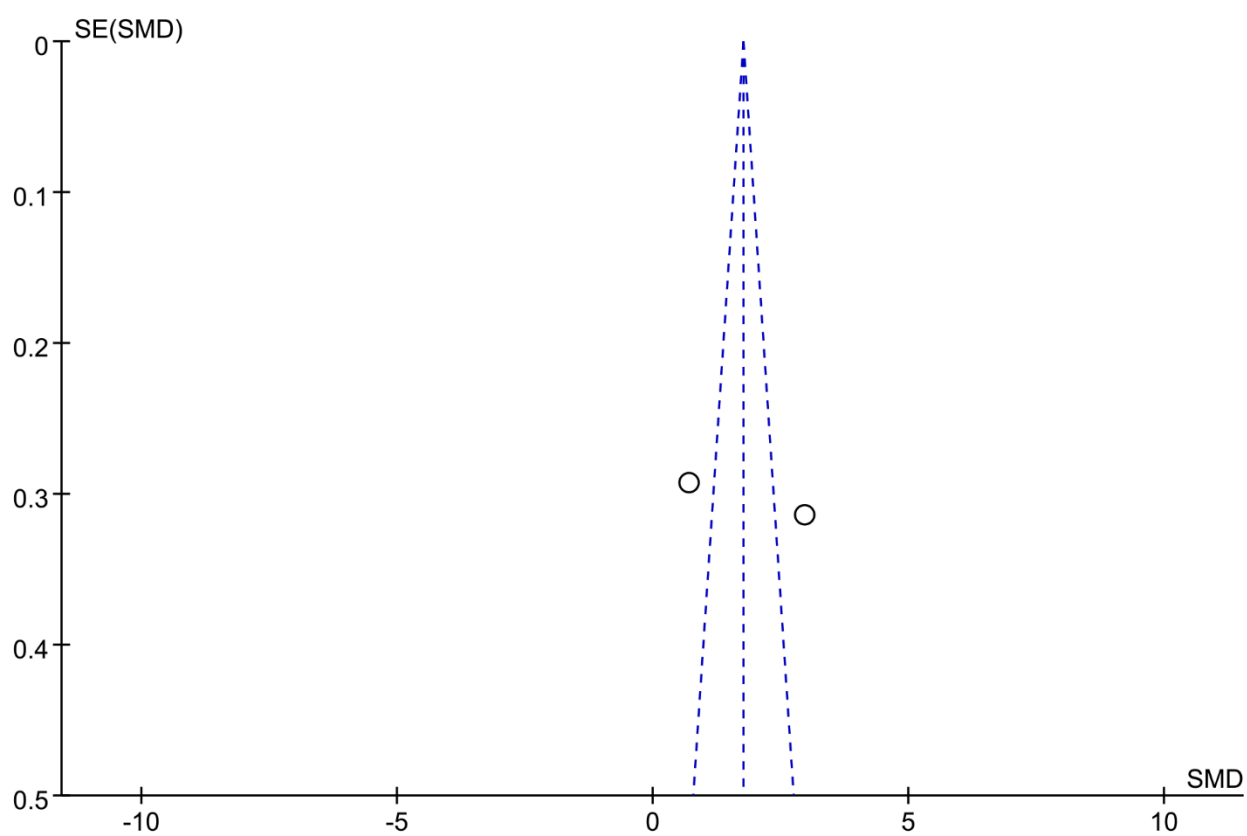

Figure S1 The funnel chart of PASI

Supplement: Supplementary Materials — PRISMA 2009 checklist: a checklist of systematic review and meta-analysis for RCT. Figure S1: the funnel chart of PASI. [file 7552546.f1.zip › Fig S1.pdf]
